# Early Post-Transplant Changes in Lipoprotein(a), Autotaxin Activity, and Lipid Profile: A Prospective Observational Study of Tacrolimus-Treated Kidney Transplant Recipients in Poland

**DOI:** 10.3390/ijms27062641

**Published:** 2026-03-13

**Authors:** Beata Bzoma, Agnieszka Kuchta, Magdalena Dzwonkowska, Daria Kazimierska, Maciej Jankowski, Alicja Dębska-Ślizień

**Affiliations:** 1 Department of Nephrology, Transplantology and Internal Medicine, Medical University of Gdańsk, University Clinical Center in Gdańsk, Smoluchowskiego 17, 80-214 Gdańsk, Poland; adeb@gumed.edu.pl; 2 Department of Clinical Chemistry, University Clinical Center in Gdańsk, Medical University of Gdańsk, Dębinki 7, 80-210 Gdańsk, Poland; dkazimierska@gumed.edu.pl (D.K.); agnieszka.kuchta@gumed.edu.pl (A.K.); maciej.jankowski@gumed.edu.pl (M.J.); 3 Central Clinical Laboratory, University Clinical Center in Gdańsk, Dębinki 7, 80-952 Gdańsk, Poland; mdzwonkowska@uck.gda.pl

**Keywords:** Lipoprotein(a), dyslipidemia, autotaxin, kidney transplantation

## Abstract

Kidney transplantation (KTx) corrects many uremia-related metabolic disturbances; however, dyslipidemia remains common in kidney transplant recipients and contributes to persistent cardiovascular risk. Lipoprotein(a) [Lp(a)] is a largely genetically determined proatherogenic lipoprotein that increases in advanced chronic kidney disease (CKD) and may decrease after restoration of renal function. Autotaxin (ATX), an enzyme involved in proinflammatory lipid signaling through the ATX–lysophosphatidic acid axis, has also been implicated in cardiovascular pathology, but its early post-transplant dynamics remain poorly characterized. In addition to quantitative lipid abnormalities, CKD is associated with high-density lipoprotein (HDL) dysfunction and reduced paraoxonase-1 (PON-1) activity; however, data on early post-transplant changes in PON-1 activity are limited. In this prospective observational study, lipid profile parameters, Lp(a) concentration, ATX activity, and PON-1 activity were assessed in 55 Caucasian patients with CKD stage 5, most of whom were dialysis-dependent, before and 2–3 weeks after KTx. All recipients received tacrolimus-based maintenance immunosuppression with corticosteroids and mycophenolate mofetil. After KTx, Lp(a) levels decreased by a median of 21% and ATX activity by 28% (both *p* < 0.001). Lp(a) and ATX showed no cross-sectional or longitudinal association either before or after transplantation, and their percentage changes were not correlated. In contrast, conventional lipid fractions increased significantly, including total cholesterol (+22%), LDL cholesterol (+27%), HDL cholesterol (+24%), and triglycerides (+55%) (all *p* < 0.001). PON-1 activity increased by approximately 13% after KTx (*p* < 0.001), and its percentage change correlated positively with the increase in HDL cholesterol. In exploratory analyses, the magnitude of Lp(a) reduction was associated with early graft function: patients with eGFR <45 mL/min/1.73 m^2^ exhibited a significantly smaller decline in Lp(a) than those with better graft function (−4.8% vs. −26.7%, *p* = 0.009). Multivariable analysis showed that demographic characteristics, body mass index, tacrolimus exposure, and post-transplant eGFR did not independently predict the magnitude of Lp(a) reduction. Tacrolimus trough concentrations and cumulative corticosteroid exposure were not associated with lipid parameters or their changes, except for a single subgroup difference in PON-1 activity of uncertain clinical significance. In summary, in the early period after KTx under tacrolimus-based immunosuppression, Lp(a) concentration and ATX activity decrease, whereas conventional lipid fractions increase and PON-1 activity improves. These changes were not associated with tacrolimus exposure or cumulative corticosteroid dose. The reduction in Lp(a) was associated with early graft function in exploratory analyses, suggesting that recovery of renal function may contribute to early post-transplant Lp(a) dynamics; however, no independent causal relationship was established, and the findings should be interpreted cautiously given the limited sample size and exploratory design. The clinical significance of these changes for long-term cardiovascular and graft outcomes requires further investigation.

## 1. Introduction

Chronic kidney disease (CKD) is associated with profound disturbances in lipid metabolism, contributing substantially to the high cardiovascular morbidity and mortality observed in this population. The characteristic lipid pattern observed in CKD—often referred to as uremic dyslipidemia—includes elevated triglyceride (TG) levels, reduced high-density lipoprotein cholesterol (HDL-C), and usually normal or only mildly increased low-density lipoprotein cholesterol (LDL-C), along with increased concentrations of lipoprotein(a) [Lp(a)] [1,2,3]. Lp(a) is a distinct LDL-like particle containing apolipoprotein(a) [apo(a)], encoded by the LPA gene and primarily synthesized in the liver [4]. Although Lp(a) concentration is highly genetically determined and largely dependent on production rate [5,6,7], kidney function plays an important role in regulating circulating Lp(a) concentration. Lp(a) concentration is consistently elevated in patients with CKD, and declining glomerular filtration rate has been associated with progressive increases in circulating Lp(a) concentrations [3,8,9,10,11]. Several studies have reported reductions in Lp(a) concentration after successful kidney transplantation (KTx); however, the magnitude, determinants, and persistence of this effect have varied substantially across cohorts [8,12,13,14,15]. Reports examining the early post-transplant phase described reductions of approximately 30–40% within the first weeks following transplantation [12,13,14,16,17,18], whereas longer-term observations indicated that Lp(a) levels frequently remain elevated compared with healthy individuals despite restoration of renal function [8,14,15].

In the general population, elevated Lp(a) is a well-established independent risk factor for cardiovascular disease and calcific aortic valve stenosis [19,20]. Importantly, increased Lp(a) concentrations have also been independently associated with a higher risk of coronary artery disease, myocardial infarction, and mortality in patients with CKD, irrespective of traditional cardiovascular risk factors [3,21,22,23,24,25]. While the precise mechanisms underlying the pathogenic effects of Lp(a) remain incompletely understood, Lp(a) retains the atherogenic properties of LDL and serves as the principal carrier of oxidized phospholipids (OxPLs), thereby promoting oxidative stress, inflammation, and endothelial dysfunction. Additional proatherogenic mechanisms include the selective transport of autotaxin (ATX), an enzyme that converts lysophosphatidylcholine (LPC) into lysophosphatidic acid (LPA), a bioactive lipid mediator involved in inflammation, fibrosis, and vascular remodeling [26,27]. Despite increasing interest in the ATX–LPA axis, its early behavior after KTx, particularly in the immediate post-transplant period, remains poorly characterized.

At the same time, kidney transplant recipients (KTRs) frequently develop de novo or worsening dyslipidemia, which perpetuates cardiovascular risk and may contribute to chronic allograft dysfunction through endothelial injury and accelerated graft vasculopathy. Dyslipidemia affects up to 60% of KTRs [21,28,29] and cardiovascular disease remains the leading cause of morbidity and mortality in this population [30].

In the early post-transplant phase, changes in lipid metabolism may reflect the combined effects of immunosuppressive therapy, including calcineurin inhibitors, mTOR inhibitors, and corticosteroids, as well as the correction of uremia following KTx [30,31,32,33,34,35].

Among calcineurin inhibitors, cyclosporine (CsA), introduced in the 1980s, exerts a pronounced hyperlipidemic effect, characterized by increases in TC, LDL-C, and TG. Available data indicate that CsA is associated with higher Lp(a) concentrations, as demonstrated in a direct comparison of KTRs receiving CsA monotherapy versus those treated with azathioprine and prednisolone [16,36]. Tacrolimus, a calcineurin inhibitor introduced more recently and currently replacing CsA in most transplant recipients worldwide, is generally associated with a milder dyslipidemic profile than CsA [37,38,39]. Its metabolic effects mainly involve moderate hypertriglyceridemia, with a relatively limited impact on total cholesterol (TC) and LDL-C levels [40,41,42,43]. However, in contrast to classical lipid parameters, early post-transplant dynamics of Lp(a) under contemporary tacrolimus-based immunosuppression remain insufficiently characterized and have not been systematically evaluated. We therefore intentionally restricted our cohort to tacrolimus-treated recipients to minimize pharmacological confounding and to better isolate the impact of renal function recovery. It should also be noted that corticosteroids, routinely administered after transplantation, contribute significantly to metabolic disturbances, including hypertriglyceridemia, impaired glucose metabolism, increased hepatic lipogenesis, and alterations in lipid regulation [31,32].

Importantly, post-transplant lipid profiles may be influenced not only by immunosuppressive exposure but also by baseline cardiovascular risk, pre-existing lipid disorders, dialysis duration, and concomitant medications, further complicating the interpretation of lipid dynamics in this population. In addition to quantitative lipid disturbances, CKD is characterized by qualitative lipoprotein dysfunction, including impaired HDL antioxidant capacity and reduced paraoxonase-1 (PON-1) activity, an HDL-associated enzyme that contributes to the antioxidative and anti-inflammatory properties of HDL [44,45]. Data regarding early post-transplant restoration of HDL functionality remains limited. The early post-transplant period represents a distinct metabolic transition characterized by abrupt restoration of glomerular filtration, changes in inflammatory status, and initiation of maintenance immunosuppression. Rapid shifts in lipid metabolism during this phase may shape subsequent cardiovascular risk trajectories. A clearer understanding of early lipid and biomarker dynamics may therefore contribute to improved cardiovascular risk stratification in kidney transplant recipients.

Taken together, KTx induces profound changes in lipid metabolism, oxidative stress, and inflammatory signaling that may influence cardiovascular risk in transplant recipients. We evaluated Lp(a), ATX, and PON-1 because these biomarkers reflect complementary aspects of lipid-related cardiovascular risk biology, encompassing atherogenic burden, inflammatory lipid signaling, and HDL-associated antioxidative capacity.

In light of these considerations, we hypothesized that restoration of renal function after KTx would be associated with an early decline in Lp(a) and ATX activity and with changes in HDL-associated antioxidative capacity reflected by increased PON-1 activity. We further explored whether these biomarker changes were associated with graft function recovery and immunosuppressive exposure. Accordingly, this study aimed to assess changes in Lp(a) concentration and ATX activity during the early post-transplant period (within the first 2–3 weeks after KTx) in tacrolimus-treated recipients. The primary objective was to evaluate early changes in Lp(a) and ATX, whereas secondary analyses examined their associations with immunosuppressive exposure—defined as mean tacrolimus trough concentration and cumulative methylprednisolone dose per kilogram of body weight—as well as lipid profile alterations, PON-1 activity, and graft function.

## 2. Results

### 2.1. Baseline Characteristics of the Study Population and Follow-Up Outcomes

#### 2.1.1. Baseline Characteristics

The study cohort consisted of 55 KTRs. The median age was 45 years (IQR 38, 57), and 58.2% of patients were male. The median BMI was 24.5 kg/m^2^ (IQR 22.5, 27.8), and 10.9% of participants were obese (BMI > 30 kg/m^2^). The most common causes of ESKD were glomerulonephritis (38.2%) and autosomal dominant polycystic kidney disease (ADPKD, 16.4%). Prior to transplantation, most patients were treated with hemodialysis (HD, 76.4%) or peritoneal dialysis (PD, 12.7%), while 10.9% underwent preemptive transplantation (PREE). Among patients undergoing renal replacement therapy (RRT), the median duration of RRT was 30.2 (IQR 17.3, 54.1) months (range 4–353 months). Donors had a median age of 47 years (IQR 33, 56), and 52.7% were male. Induction therapy was administered in 94.5% of cases (Thymoglobulin^®^/Grafalon^®^ or Simulect^®^), and all patients received tacrolimus-based maintenance immunosuppression. The median mean tacrolimus trough concentration (C0) from three trough measurements was 12.7 ng/mL (IQR 11.27, 15) and the median cumulative methylprednisolone dose/kg over the first 14 days post-transplant was 26.7 mg/kg (24.3, 33.3).

2–3 weeks after KTx, median serum creatinine was 1.68 mg/dL, median eGFR CKD-EPI was 43 mL/min/1.73 m^2^.

#### 2.1.2. Follow-Up Outcomes

Acute rejection (AR) occurred in 5.4% of patients, delayed graft function (DGF) in 14.5%, and post-transplant diabetes mellitus (PTDM) in 14.5%. One patient (1.8%) died from sepsis 14 months after KTx. Graft loss occurred in two patients (3.6%), both due to BK virus nephropathy, at 2.5 and 3 years after KTx. The characteristics of the study population are presented in Table 1.

### 2.2. Lp(a) and ATX Dynamics Following KTx

**Figure 1:**
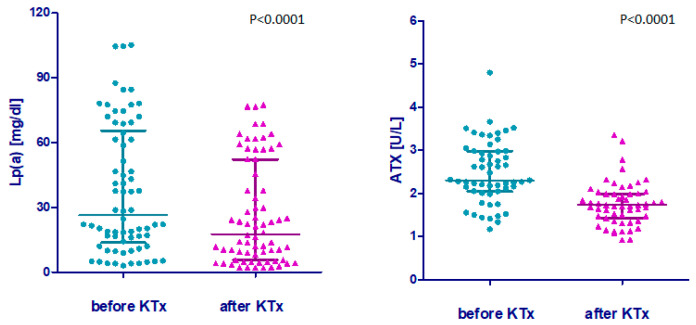
Lp(a) concentration and ATX activity before and after KTx. Results are presented as single points, median and interquartile range; significance of differences was assessed by the Wilcoxon test; Lp(a)—lipoprotein(a), ATX—autotaxin.

**Figure 2:**
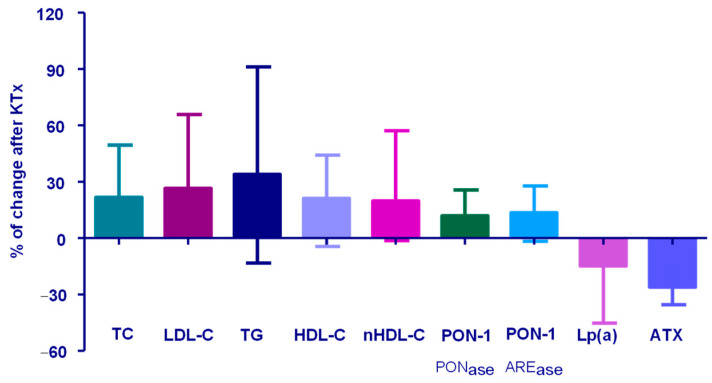
Changes in lipid-related parameters after KTx. Results are presented as median with interquartile range. Abbreviations: TC—total cholesterol; LDL-C—low-density lipoprotein cholesterol; HDL-C—high-density lipoprotein cholesterol; TG—triglycerides; nHDL-C—non-HDL cholesterol; PON-1_PONase_—paraoxonase-1 activity toward paraoxon; PON-1_AREase_—paraoxonase-1 activity toward phenyl acetate; Lp(a)—lipoprotein(a); ATX—autotaxin.

The results demonstrated a significant reduction in Lp(a) concentration and ATX activity after KTx. Lp(a) concentration decreased by 21%, and ATX activity decreased by 28% (Figure 1 and Figure 2, Table 2). However, Lp(a) concentration did not correlate with ATX activity either before (ρ = 0.024, 95% CI −0.250 to 0.295; *p* = 0.578) or after KTx (ρ = 0.206, 95% CI −0.070 to 0.453, *p* = 0.146). Similarly, the percentage changes in Lp(a) and ATX following transplantation showed no significant correlation (ρ = −0.050, 95% CI −0.326 to 0.227; *p* = 0.741).

### 2.3. Lipid-Related Parameters Before and After KTx

The values of the analyzed lipid-related parameters before and after KTx are presented in Table 2 and Figure 2. In contrast to Lp(a) and ATX, TC, LDL-C, HDL-C, and TG levels increased following KTx. We observed a significant increase in TC (22%), LDL-C (27%), and HDL-C (24%). The largest increase was observed in TG concentration, with a median increase of 55% (Table 2, Figure 2).

In addition, the activity of the antioxidative enzyme PON-1, measured toward both paraoxon (PON-1_PONase_) and phenyl acetate (PON-1_AREase_), increased by an average of 13% after KTx. HDL-C concentration was not correlated with paraoxonase-1 activity against either substrate before KTx (ρ = 0.102, 95% CI −0.175 to 0.036; *p* = 0.450 for PON-1_PONase_; ρ = 0.303, 95% CI −0.033 to 0.532; *p* = 0.070 for PON-1_AREase_) or afterwards (ρ = −0.086, 95% CI −0.1914 to 0.350; *p* = 0.392 for PON-1_PONase_; ρ = −0.119, 95% CI −0.382 to 0.162; *p* = 0.533 for PON-1_AREase_). However, the percentage changes in both measures of PON-1 activity were positively correlated with changes in HDL-C concentration (ρ = 0.622, 95% CI 0.420 to 0.765; *p* < 0.001 for PON-1_PONase_; ρ = 0.462, 95% CI 0.213 to 0.653; *p* < 0.001 for PON-1_AREase_).

### 2.4. Association of Lp(a) Concentration and ATX Activity with Early Post-Transplant Kidney Function

A modest inverse correlation was observed between the percentage change in Lp(a) concentration and post-transplant eGFR (Spearman’s ρ = −0.279, 95% CI −0.54 to −0.05; *p* = 0.039). This association should be interpreted as exploratory. In contrast, no significant association was found between the percentage change in ATX activity and eGFR (ρ = 0.089, 95% CI −0.190 to 0.356; *p* = 0.518).

At 2–3 weeks post-transplant, 27 patients (49%) had an eGFR below 45 mL/min/1.73 m^2^, while the remaining patients had an eGFR ≥45 mL/min/1.73 m^2^. The median percentage reduction in Lp(a) concentration was significantly smaller in patients with eGFR <45 mL/min/1.73 m^2^ compared with those with eGFR ≥45 mL/min/1.73 m^2^ (−4.8% vs. −26.7%; *p* = 0.009). The median absolute reduction in Lp(a) was also greater in patients with eGFR ≥45 mL/min/1.73 m^2^ (−9.9 mg/dL vs. −0.7 mg/dL; *p* < 0.01). No such differences were observed for ATX activity (−27.1% vs. −29.0; *p* = 0.75).

### 2.5. Multivariable Regression Analysis of Predictors of Lp(a) Change

To determine whether demographic or transplant-related factors influence the magnitude of early post-transplant reductions in Lp(a), we performed a multiple linear regression analysis including age, BMI, tacrolimus exposure, and post-transplant eGFR (CKD-EPI).

The overall model did not reach statistical significance (F(4,50) = 0.86, *p* = 0.49), with low explanatory power (R^2^ = 0.06; adjusted R^2^ = −0.01). None of the variables independently predicted the percentage reduction in Lp(a), with standardized beta coefficients ranging from −0.12 to +0.14. No independent predictors of percentage Lp(a) change were identified in the exploratory regression model. However, given the limited sample size and low explanatory power of the model, the analysis may have been underpowered to detect modest independent effects.

### 2.6. Blood Tacrolimus Concentration and Cumulative Methylprednisolone Dose in Relation to Lipid Parameters

No significant correlations were observed between mean tacrolimus blood concentration or the cumulative dose of methylprednisolone and any of the analyzed lipid-related parameters (TC, LDL-C, HDL-C, TG, nHDL-C, PON-1_PONase_, PON-1_AREase_, Lp(a), ATX), nor with their percentage changes following KTx. There were no significant differences in the changes pre- vs. post-KTx of the analyzed lipid in those with cumulative steroid doses above and below 30 mg/kg, or those with mean tacrolimus trough blood concentration above and below 15 ng/mL, with the exception of the percentage change in PON-1_PONase_ activity pre- versus post-KTx, which was significantly greater in patients with mean tacrolimus trough concentration >15 ng/mL (median 26.5, IQR 14–30.8) compared with those with concentration ≤15 ng/mL (median 10.0, IQR 3.0–20.0; Mann–Whitney U test, *p* = 0.012). In contrast, no significant differences were observed when PON-1 activity was assessed using the AREase method.

### 2.7. Comparison of Lipid-Related Parameters According to Patients’ Clinical Characteristics

There were no significant differences in the changes pre- vs. post-KTx of the analyzed lipid parameters (TC, LDL-C, HDL-C, TG, nHDL-C, PON-1_PONase_, PON-1_AREase_, Lp(a), ATX) between patients with and without DGF. Furthermore, no significant differences were observed between patients with and without obesity, or between older (≥60 years) and younger (<60 years) patients. No significant differences in pre- to post-KTx changes in lipid parameters were observed when patients were stratified by pre-transplant dialysis modality (HD vs. PD, PREE patients were excluded from the comparison).

## 3. Discussion

Lp(a) is a well-established independent risk factor for cardiovascular disease, which remains the leading cause of morbidity and mortality among KTRs. In this prospective observational study, we evaluated early changes in Lp(a) concentration and ATX activity, and HDL-associated PON-1 activity in patients with ESKD undergoing KTx under tacrolimus-based immunosuppression.

The main findings are threefold. First, both Lp(a) levels and ATX activity decreased significantly within 2–3 weeks after KTx. Second, despite parallel reductions, Lp(a) did not correlate with ATX either before or after KTx, and their changes were not associated, suggesting potentially distinct regulation in the early post-transplant period.

Third, conventional lipid fractions increased markedly in the early post-transplant period, whereas PON-1 activity improved and its change was positively related to the rise in HDL-C. Notably, the magnitude of Lp(a) reduction was associated with early graft function, while mean tacrolimus trough blood levels and cumulative steroid exposure were not.

Consistent with prior reports, Lp(a) levels decline rapidly after successful KTx, with substantial reductions occurring within the first 1–2 weeks, in parallel with early graft function recovery. In our study, the median reduction in Lp(a) levels was 20.7%. Importantly, essentially all earlier studies demonstrating a reduction in Lp(a) after KTx were conducted during the CsA era. Our findings extend these observations to a contemporary cohort receiving tacrolimus-based immunosuppression and provide novel data on early Lp(a) dynamics under current immunosuppressive strategies. However, the study was not designed to compare immunosuppressive regimens, and therefore no conclusions regarding drug-specific effects can be drawn.

Approximately half of the recipients had an eGFR <45 mL/min/1.73 m^2^ at 2–3 weeks after KTx. This cutoff was prespecified based on CKD staging (threshold between stages 3a and 3b), reflecting clinically meaningful differences in graft function and metabolic risk. Patients with lower eGFR exhibited a markedly smaller reduction in Lp(a) (approximately 5% vs. 27%). However, post-transplant eGFR did not independently predict Lp(a) change in multivariable analysis when modeled as a continuous variable. The discrepancy between categorical and continuous analyses may indicate a threshold effect, whereby restoration of renal function beyond a certain level is associated with a greater decline in Lp(a), while incremental improvements confer limited additional impact. Given the limited sample size and restricted variability in early post-transplant eGFR, this interpretation should be considered hypothesis-generating.

In our study, no differences in Lp(a) levels or ATX activity were observed between recipients with and without DGF. However, this finding should be interpreted cautiously, as only eight patients developed DGF, resulting in limited power to detect small-to-moderate differences. Moreover, DGF is defined by dialysis requirement in the first post-transplant week, whereas our biochemical sampling occurred 2–3 weeks after transplantation, a time point when graft function in most patients had already recovered or stabilized.

The observed decrease in ATX activity after KTx may be related to improved renal function and associated changes in inflammatory and proatherogenic pathways. As ATX generates lysophosphatidic acid, a bioactive lipid involved in fibrosis, vascular remodeling, and endothelial dysfunction, its reduction may reflect alterations in lipid-mediated cardiovascular and metabolic signaling pathways in transplant recipients. However, the clinical significance of this finding remains uncertain. It should also be noted that glucocorticoid administration has been shown to reduce circulating ATX antigen levels. This effect is likely mediated by decreased ATX mRNA expression in adipose tissue following steroid treatment [46]. The lack of association between steroid exposure and autotaxin activity in our study may reflect the assessment of enzymatic activity rather than antigen levels, as well as the short and relatively uniform steroid exposure in the early post-transplant period. Importantly, prospective data describing early ATX activity changes after KTx remain scarce. Our findings therefore extend current understanding by demonstrating that ATX activity declines rapidly after KTx in a tacrolimus-treated cohort.

While both Lp(a) levels and ATX activity declined after KTx, Lp(a) did not correlate with ATX activity either before (*p* = 0.578) or after KTx (*p* = 0.146). Similarly, the percentage changes in Lp(a) and ATX following KTx showed no significant correlation (*p* = 0.741). In advanced CKD, a shared proinflammatory environment may act as a common upstream driver of both Lp(a) and ATX. After KTx, removal of this stimulus may result in parallel reductions in both parameters while revealing their independent regulation. In contrast, conventional lipid fractions, including TC, LDL-C, HDL-C, TG, and nHDL-C, increased after KTx. No significant associations were observed between lipid parameters and tacrolimus exposure or cumulative methylprednisolone dose. The observed post-transplant increase in conventional lipid fractions may, at least in part, reflect recovery from the uremic state, resolution of inflammation, and normalization of metabolic pathways, rather than being solely attributable to the direct effects of immunosuppressive therapy.

At the same time, PON-1 activity, assessed toward both paraoxon and phenyl acetate, increased significantly, suggesting a potential improvement in HDL-related antioxidative properties; however, this represents an indirect inference based on PON-1 activity and does not directly assess HDL function or oxidative stress.

It has been reported that patients undergoing continuous ambulatory peritoneal dialysis (PD) prior to transplantation exhibit higher Lp(a) levels compared with those on HD, which may influence post-transplant Lp(a) levels [10,14,47]. In contrast, our study did not reveal any differences in Lp(a), PON-1, or other lipid parameters—either before or after KTx, or in their post-transplant changes—between patients previously treated with HD and those managed with PD. Although ATX activity after KTx was numerically higher in recipients previously treated with PD, no differences were observed in ATX levels before KTx or in post-transplant changes. This finding should therefore be interpreted with caution and may represent a spurious association.

Although elevated Lp(a) is an independent cardiovascular risk factor in both the general population [12,13] and patients with CKD [3], and KTx reduces Lp(a) levels in most recipients, it remains unclear whether these post-transplant reductions translate into clinically meaningful cardiovascular risk reduction. According to current guidelines, measurement of Lp(a) should be considered at least once in a lifetime for this population [48], or if there is a family history of premature atherosclerotic cardiovascular disease (ASCVD) or unexplained personal ASCVD, as in the general population [49]. Elevated Lp(a) levels should prompt more intensive LDL-C lowering strategies and closer cardiovascular surveillance.

Beyond its established role in cardiovascular risk, Lp(a) may also be relevant to kidney allograft outcomes through its proinflammatory and proatherogenic effects on endothelial and microvascular injury. Persistently elevated Lp(a) after transplantation could potentially contribute to progressive graft dysfunction, although evidence remains limited [50]. As the present study was restricted to the early post-transplant period, we could not assess associations between early Lp(a) dynamics and long-term graft outcomes, which require further longitudinal investigation.

Future studies should extend follow-up beyond the early post-transplant period, incorporate genetic analyses of LPA and PON-1 polymorphisms, and evaluate associations with clinically relevant outcomes, including cardiovascular events and graft survival.

This study has several limitations. The modest sample size (*n* = 55), single-center design, predominance of deceased-donor transplants, and predominantly Caucasian cohort may limit statistical power and generalizability. Strict inclusion criteria, exclusion of cyclosporine-treated patients and those receiving lipid-lowering therapy, and logistical constraints related to sample processing may have influenced study selection.

Clinical heterogeneity and variability in pre-transplant status (including duration of dialysis therapy, CKD progression, baseline lipid profile, comorbidities, and inflammatory status), together with unmeasured genetic, dietary, and pharmacological factors, including concomitant medications administered during the early post-transplant period, represent potential sources of residual confounding. Although paired within-subject analyses reduce between-patient variability, perioperative metabolic changes may also have contributed to the observed biomarker dynamics.

The absence of a non-transplanted control group and the short follow-up (2–3 weeks) limit assessment of long-term cardiovascular and graft outcomes; therefore, the findings should be interpreted as mechanistic and hypothesis-generating.

## 4. Materials and Methods

### 4.1. Patients

**Figure 3:**
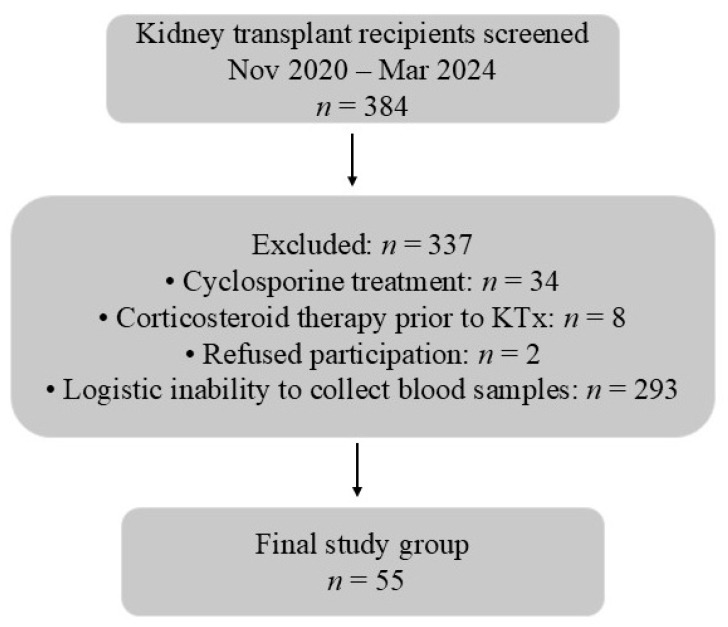
Flowchart of the study.

We prospectively analyzed lipid profile parameters, Lp(a) concentrations, and ATX activity in a cohort of Caucasian patients before and 2–3 weeks after KTx. The study included 55 patients with CKD5, most of whom were CKD5D, who underwent KTx at the Department of Nephrology, Transplantology, and Internal Diseases, Medical University of Gdańsk, between November 2020 and March 2024. Eligible patients were screened consecutively, and those who met the inclusion criteria were enrolled when sampling and logistical conditions permitted. During this period, a total of 384 patients underwent KTx at our center. The inclusion and exclusion criteria are listed below and the study flowchart is presented in Figure 3. The study cohort consisted of 23 women and 32 men, with a median age of 45 years (range: 27–70 years). All patients received tacrolimus, methylprednisolone, and MMF as their immunosuppressive regimen, with induction therapy administered in almost all cases. None of the participants received lipid-lowering medication during the study period. Only one patient received a kidney from a living donor; the remainder underwent deceased donor transplantation (donation after brain death—DBD). Baseline clinical and transplant-related characteristics are summarized in Table 1.

#### 4.1.1. Inclusion and Exclusion Criteria

Inclusion Criteria:

- Adult patients (≥18 years old) who underwent KTx at the Gdańsk Transplantation Center between November 2020 and March 2024.
- Signed informed consent for participation in the prospective observational study.

Exclusion Criteria:

- CsA as a component of the immunosuppression regimen.
- Systemic corticosteroid therapy prior to transplantation, defined as the use of systemic corticosteroids at the time of KTx or within the preceding 3 months.
- Refusal to participate in the study.
- Inability to perform laboratory tests required for the study due to timing/logistics *.

* Most KTx at our center are performed using organs from deceased donors and recipients are transported to the donor’s transplant center on short notice, often at any time of day or night. Due to the unpredictable timing of transplant admissions and the limited availability of study personnel outside standard working hours, it was not always possible to perform the required pre-transplant blood processing. Consequently, patients for whom proper sample handling could not be ensured were excluded from the study. The inability to obtain study samples was unrelated to patient clinical characteristics and resulted solely from logistical constraints; therefore, study inclusion was independent of patient clinical status and transplant-related factors.

#### 4.1.2. Definition of Comorbidities and Clinical Conditions

Comorbidities and transplant-related complications were identified based on a comprehensive review of medical records, transplant documentation, and laboratory/imaging results.

- Charlson Comorbidity Index (CCI): Calculated using the original scoring system [51], which assigns weighted points to comorbid conditions (e.g., cardiovascular disease, chronic pulmonary disease, malignancy, liver disease) based on the patient’s documented medical history at the time of KTx.
- AR-Acute Rejection: Defined as a clinically suspected episode of immunologic graft injury, characterized by a ≥25% increase in serum creatinine from baseline in the absence of other identifiable causes (e.g., dehydration, infection, urinary tract obstruction, or nephrotoxic levels of calcineurin inhibitors). Due to the observational nature of this study and institutional practice patterns, biopsy confirmation was not consistently performed. The diagnosis was based on clinical signs (e.g., rising creatinine, graft tenderness, reduced urine output), an elevated Resistance Index (RI) on Doppler ultrasound, and the patient’s response to anti-rejection treatment (high-dose corticosteroids). All episodes of AR were reviewed retrospectively using medication records. Episodes of suspected AR were treated with high-dose intravenous methylprednisolone boluses and were incorporated into the calculation of cumulative corticosteroid exposure (total methylprednisolone dose per kilogram of body weight).
- DGF-Delayed graft function: Defined as the requirement for dialysis during the first 7 days following transplantation.
- PTDM-Post-transplant diabetes mellitus: Defined as the requirement for pharmacologic therapy at least three months after transplantation or diagnosis based on the results of an oral glucose tolerance test (OGTT). The 3-month threshold was applied to avoid misclassification due to transient post-transplant hyperglycemia in the early post-transplant period. PTDM was assessed during longitudinal follow-up and was not part of the early post-transplant endpoints.
- Graft Loss: Defined as return to chronic dialysis, graft nephrectomy.

### 4.2. Laboratory Measurements

We analyzed laboratory parameters at two time points: before KTx and 2–3 weeks after the procedure.

Peripheral fasting blood samples were collected following an 8 h fast, which was confirmed prior to sampling. Pre-transplant samples were collected 2–8 h before transplantation, and in dialysis-dependent patients the last dialysis session was typically performed within 24 h prior to transplantation. Post-transplant samples were collected under comparable clinical conditions either during hospitalization or at the first outpatient visit after hospital discharge.

Serum was separated by centrifugation at 1000× *g* for 15 min and stored at −80 °C until analysis. The maximum storage duration did not exceed 6 months. All specimens were coded prior to laboratory analysis. To minimize inter-assay variability, pre- and post-transplant samples obtained from the same patient were analyzed within the same assay series whenever possible. Laboratory personnel were blinded to clinical outcomes and had access only to coded identifiers and pairing information.

TC, LDL-C, HDL-C and TG were measured using standard direct enzymatic colorimetric assays (Wiener Lab, Warsaw, Poland).

Lp(a) concentration was determined with a commercially available immunoturbidimetric assay (Randox, Crumlin, UK). The assay was calibrated using a five-point Randox calibrator (Randox, Crumlin, UK) and is designed to minimize interference related to apolipoprotein(a) size heterogeneity, thereby reducing analytical bias arising from different apo(a) isoforms. Measurements were performed according to the manufacturer’s instructions, with internal quality controls applied.

ATX activity was assessed based on the hydrolysis rate of 1-myristoyl-sn-glycero-3-phosphocholine [52], quantified by measuring choline as the reaction product using a colorimetric assay (Merck, Warsaw, Poland). One unit (U) of ATX activity was defined as the amount of enzyme that converts 1 μmol of substrate per minute at 37 °C. ATX activity was measured in duplicate, within the same assay run, and inter-assay variability was ±5%.

Paraoxonase-1 (PON-1) activity was determined as paraoxonase activity using paraoxon (PON-1_PONase_; U/L) and as arylesterase activity using phenyl acetate (PON-1_AREase_; kU/L) in a calcium-containing buffer under standardized conditions. Reaction rates were recorded spectrophotometrically and converted to activity units using established extinction coefficients. One unit of PON-1_PONase_ activity was defined as the amount of enzyme that converts 1 µmol of diethyl paraoxon into p-nitrophenol and diethyl phosphate per minute at 37 °C. One unit of PON-1_AREase_ activity was defined as the amount of enzyme that converts 1 µmol of phenyl acetate into phenol and acetone per minute at 37 °C. PON-1_PONase_ and PON-1_AREase_ activities were measured in duplicate within the same assay run, and inter-assay variability was ±4% and ±6% respectively.

Serum creatinine was measured using an IDMS-traceable enzymatic assay on an Abbott Alinity analyzer, with duplicate measurements and two-level internal quality control.

eGFR was calculated using the CKD-EPI 2009 equation, which was the standard method in our clinical practice at the time of the study. Measurements were obtained 2–3 weeks after transplantation, when kidney function was considered clinically stable.

Tacrolimus trough concentration (C0) was determined in whole-blood samples collected in the fasting state immediately before the morning dose as part of routine therapeutic drug monitoring. Blood samples were processed according to standard laboratory procedures, and tacrolimus concentrations were expressed in ng/mL.

Lipid profile, Lp(a), ATX, PON-1 activity, and serum creatinine were measured from the same blood sample at each time point, whereas tacrolimus exposure was assessed as the mean trough concentration calculated from three trough level measurments during the first 2 weeks after KTx.

### 4.3. Statistical Analysis

Statistical analyses were performed using STATISTICA software version 10 (StatSoft, Warsaw, Poland) and GraphPad Prism version 8.0 (GraphPad Software, San Diego, CA, USA). The Shapiro–Wilk test was used to assess the normality of data distribution. As most variables did not follow a normal distribution, continuous data are presented as medians with interquartile ranges (25th–75th percentiles). Between-group comparisons were performed using the nonparametric Mann–Whitney U test. Categorical variables were compared using Pearson’s chi-square test. Spearman’s rank correlation coefficients (ρ) were calculated to assess associations between variables, and two-tailed *p*-values are reported. In addition, 95% confidence intervals for Spearman’s ρ were calculated using Fisher’s z transformation to provide estimates of effect size precision. Paired comparisons before and after KTx were analyzed using the Wilcoxon signed-rank test. Multivariable linear regression was used to assess independent predictors of the percentage change in Lp(a). To minimize overfitting, four prespecified covariates—age, BMI, tacrolimus trough concentration, and post-transplant eGFR—were entered into the model. Model performance was evaluated using R^2^, adjusted R^2^, and the F-test. A *p*-value < 0.05 was considered statistically significant. The study was designed as a prospective exploratory investigation. The primary outcomes were early post-transplant changes in Lp(a) and ATX activity. All additional analyses (including biomarker associations, subgroup comparisons, and correlations with eGFR or immunosuppressive exposure) were predefined as secondary and exploratory; therefore, no formal correction for multiple comparisons was applied and these results should be interpreted cautiously. A formal a priori sample size calculation was not performed due to the lack of reliable effect size estimates for early post-transplant changes in Lp(a) and ATX activity under tacrolimus-based immunosuppression. Analyses were conducted using complete-case methodology, as the proportion of missing data was minimal and did not require imputation.

The tacrolimus cutoff (>15 ng/mL) corresponded to concentrations exceeding the recommended target trough range (12–15 ng/mL) during the first month after KTx according to national clinical recommendations [53].

The cumulative methylprednisolone dose cutoff (30 mg/kg) was selected based on the distribution of values in the study cohort (median 26.7 mg/kg, IQR 24.3–33.3 mg/kg) to identify patients with higher early corticosteroid exposure.

## 5. Conclusions

KTx is associated with early alterations in lipid metabolism within the first 2–3 weeks after surgery. In this tacrolimus-treated cohort, conventional lipid fractions (TC, LDL-C, HDL-C, TG, and nHDL-C) increased, whereas Lp(a) concentration and ATX activity declined and PON-1 activity increased. These changes were not associated with tacrolimus exposure or cumulative corticosteroid dose, suggesting that modulation of Lp(a), ATX, and PON-1 may primarily reflect recovery of renal function rather than direct metabolic effects of immunosuppressive therapy. Given the exploratory design and moderate sample size, the findings should be considered hypothesis-generating. Larger prospective studies with longer follow-up are required to determine the persistence of these changes and their impact on long-term cardiovascular and graft outcomes.

## Figures and Tables

**Table 1:** Baseline clinical and transplant characteristics of the study population and follow-up outcomes.

Variable	*n* = 55
Age [years]—median (IQR); range	45 (38–57); 27–70
Age ≥ 60—*n* (%)	11 (20)
BMI [kg/m^2^] median (IQR); range	24.5 (22.5–27.8); 18–34
BMI > 30 kg/m^2^—*n* (%)	6 (11)
Gender—male *n* (%)	32 (58)
2nd and 3rd KTx—*n* (%)	8 (14.5)
Dialysis modality before KTx—*n* (%)	
HD	42 (76)
PD	7 (13)
PREE	6 (11)
Pretransplant renal replacement therapy time [months]—median (IQR); range	30.2 (17.3–54.1); 4–353
Charlson Index—median (IQR); range	2 (2–3); 2–5
MM—median (IQR); range	3 (2–4); 0–6
Cause of ESKD—*n* (%)	
GN	21 (38.2)
ADPKD	9 (16.4)
HN	6 (10.9)
IN	4 (7.3)
DM	2 (3.6)
Not known	10 (18.2)
Other	3 (5.4)
Donors’ age—median (IQR); range	47 (33–56); 14–72
Donors’ gender—male *n* (%)	29 (52.7)
WIT [min]—median (IQR); range	34 (29–43); 21–69
CIT [min]—median (IQR); range	874 (680–1388); 170–2195
IND use (Thymoglobuline^®^/Grafalon^®^ + basiliximab Simulect^®^) *n*(%)	35 + 17 = 52 (94.5)
Mean tacrolimus blood trough concentration [ng/mL] during the first 2 weeks—median (IQR); range	12.7 (11.27–15); 7.1–22.8
Mean tacrolimus blood trough concentration during the first 2 weeks ≥ 15—*n* (%)	14 (25)
Two-week cumulative methylprednisolone dose [mg/kg]—median (IQR); range	26.7 (24.3–33.3); 18.8–54
SCC 2–3 weeks after KTx [mg/dL]—median (IQR); range	1.68 (1.12–1.99); 0.59–2.99
eGFR CKD-EPI 2–3 weeks after KTx [mL/min/1.73 m^2^] median (IQR); range	43.0 (34.4–62.0); 17–126.5
Post-Tx eGFR CKD-EPI ≥ 45 [mL/min/1.73 m^2^]—*n* (%)	27 (49)
Post-Tx eGFR CKD-EPI ≥ 60 [mL/min/1.73 m^2^]—*n* (%)	16 (29)
Duration of hospitalization after KTx [days]—median (IQR); range	16 (13–21); 9–44
AR—*n* (%)	3 (5.4)
DGF—*n* (%)	8 (14.5)
PTDM—*n* (%)	8 (14.5)
Deaths—*n* (%)	1 (1.8)
Graft loss—*n* (%)	2 (3.6)

Abbreviations: ADPKD—Autosomal Dominant Polycystic Kidney Disease, AR—acute rejection, BMI—body mass index, CIT—cold ischemia time, DGF—delayed graft function, eGFR CKD-EPI—estimated glomerular filtration rate calculated using the Chronic Kidney Disease Epidemiology Collaboration equation, GN—glomerulonephritis, HD—hemodialysis, HN—hypertensive nephropathy, IN—interstitial nephritis, IND—induction, IQR—interquartile range, KTx—kidney transplantation, MM—number of mismatches, PD—peritoneal dialysis, PREE—preemptive transplantation, PTDM—post-transplant diabetes mellitus, SCC—serum creatinine concentration, WIT—warm ischemia time.

**Table 2:** Lipid-related parameters before and after KTx.

Parameter	Before KTx	After KTx	% Change	*p* Value
Total cholesterol, mg/dL	180 (166–224)	247 (194–310)	21.7 (12.4–34.9)	<0.001
LDL cholesterol, mg/dL	76 (63–106)	101 (84–136)	26.5 (14.2–41.8)	<0.001
HDL cholesterol, mg/dL	36 (29–44)	44 (36–54)	23.5 (11.6–38.9)	<0.001
Triglycerides, mg/dL	120 (94–155)	176 (113–248)	55.2 (32.1–84.7)	<0.001
Non-HDL cholesterol, mg/dL	146 (116–193)	190 (161–248)	18.9 (9.7–33.5)	<0.001
PON-1 paraoxonase activity, U/L	51 (34–97)	53 (37–106)	13.2 (4.5–28.6)	<0.001
PON-1 arylesterase activity, kU/L	23 (14–36)	27 (18–35)	13.6 (5.8–26.4)	<0.001
Lipoprotein(a) concentration, mg/dL	20 (10–47)	12 (5–25)	−20.7 (−35.4–−6.1)	<0.001
Autotaxin activity, U/L	2.3 (2.0–3.0)	1.7 (1.4–1.9)	−28.3 (−39.6–−17.5)	<0.001

Data are presented as median (interquartile range). Percentage change is expressed as median (interquartile range) and represents the relative difference between post-transplant and pre-transplant values. Comparisons between pre- and post-transplant measurements were performed using the Wilcoxon signed-rank test. Abbreviations: HDL—high-density lipoprotein; LDL—low-density lipoprotein; PON-1—paraoxonase-1; KTx—kidney transplantation.

## Data Availability

The data presented in this study are available from the corresponding author upon reasonable request.

## Abbreviations

ADPKD—Autosomal Dominant Polycystic Kidney Disease, apo(a)—apolipoprotein(a), AR—Acute Rejection, ASCVD—Atherosclerotic Cardiovascular Disease, ATX—Autotaxin, BMI—Body Mass Index, CCI—Charlson Comorbidity Index, CETP—Cholesteryl Ester Transfer Protein, CKD—Chronic Kidney Disease, CKD5—Chronic Kidney Disease stage 5, CKD5D—Dialysis-dependent Chronic Kidney Disease stage 5, C0—Tacrolimus trough concentration, CIT—Cold Ischemia Time, CsA—Cyclosporine A, CVD—Cardiovascular Disease, CYP7A1—Cholesterol 7α-hydroxylase, DBD—Donation after Brain Death, DGF—Delayed Graft Function, eGFR CKD-EPI—estimated glomerular filtration rate calculated using the Chronic Kidney Disease Epidemiology Collaboration equation, ESKD—End-Stage Kidney Disease, GN—Glomerulonephritis, HD—Hemodialysis, HDL-C—High-Density Lipoprotein Cholesterol, HN—Hypertensive Nephropathy, IDMS—Isotope Dilution Mass Spectrometry, IN—Interstitial Nephritis, IND—Induction Therapy, IQR—Interquartile Range, KTRs—Kidney Transplant Recipients, KTx—Kidney Transplantation, LPA—Lysophosphatidic Acid, LPC—Lysophosphatidylcholine, LDL-C—Low-Density Lipoprotein Cholesterol, Lp(a)—Lipoprotein(a), LPL—Lipoprotein Lipase, MM—HLA mismatches, mTORi—Mammalian Target of Rapamycin inhibitors, nHDL-C—Non-HDL Cholesterol, OGTT—Oral Glucose Tolerance Test, OxPLs—Oxidized Phospholipids, PD—Peritoneal Dialysis, PON-1—Paraoxonase-1, PON-1_AREase_—paraoxonase-1 arylesterase activity toward phenyl acetate, PON-1_PONase_—paraoxonase-1 paraoxonase activity toward paraoxon, PREE—Preemptive Kidney Transplantation, PTDM—Post-Transplant Diabetes Mellitus, RI—Resistance Index, RRT—Renal Replacement Therapy, SCC—Serum Creatinine Concentration, TC—Total Cholesterol, TG—Triglycerides, WIT—Warm Ischemia Time.
